# Supplementary material for: Resolving the full spectrum of human genome variation using Linked-Reads
Source: Genome Res. 2019 Apr;29(4):635–45. doi: 10.1101/gr.234443.118 (PMC6442396; doi:10.1101/gr.234443.118)
Supplement: Supplemental Material [file supp_gr.234443.118_Supplemental_longranger-2.2.zip › longranger-2.2.tar.bz2!/lr2.2-public/lib/stan/cmdstan/stan/doc/api/html/stanc__helper_8hpp_source.html]

Stan: src/stan/command/stanc\_helper.hpp Source File


|  |  |
| --- | --- |
|  | Stan  2.5.0  probability, sampling & optimization |


- Main Page
- Related Pages
- Namespaces
- Classes
- Files

- File List
- File Members

All Classes Namespaces Files Functions Variables Typedefs Enumerations Enumerator Friends Macros Pages

- src
- stan
- command

stanc\_helper.hpp

Go to the documentation of this file.

1 #include <stan/version.hpp>

2 #include <stan/gm/compiler.hpp>

3

4 #include <exception>

5 #include <fstream>

6 #include <iostream>

7 #include <stdexcept>

8 #include <string>

9

10 #include <stan/io/cmd\_line.hpp>

11

12 void print\_version(std::ostream\* out\_stream) {

13  if (!out\_stream) return;

14  \*out\_stream << "stanc version "

15  << stan::MAJOR\_VERSION

16  << "."

17  << stan::MINOR\_VERSION

18  << "."

19  << stan::PATCH\_VERSION

20  << std::endl;

21 }

22

26 void print\_stanc\_help(std::ostream\* out\_stream) {

27  using stan::io::print\_help\_option;

28

29  if (!out\_stream) return;

30

31  \*out\_stream << std::endl;

32  print\_version(out\_stream);

33  \*out\_stream << std::endl;

34

35  \*out\_stream << "USAGE: " << "stanc [options] <model\_file>" << std::endl;

36  \*out\_stream << std::endl;

37

38  \*out\_stream << "OPTIONS:" << std::endl;

39  \*out\_stream << std::endl;

40

41  print\_help\_option(out\_stream,"help","","Display this information");

42

43  print\_help\_option(out\_stream,"version","","Display stanc version number");

44

45  print\_help\_option(out\_stream,"name","string",

46  "Model name",

47  "default = \"$model\_filename\_model\"");

48

49  print\_help\_option(out\_stream,"o","file",

50  "Output file for generated C++ code",

51  "default = \"$name.cpp\"");

52

53 }

54

55 void delete\_file(std::ostream\* err\_stream,

56  const std::string& file\_name) {

57  int deleted = std::remove(file\_name.c\_str());

58  if (deleted != 0 && file\_name.size() > 0)

59  if (err\_stream)

60  std::cerr << "Could not remove output file=" << file\_name

61  << std::endl;

62 }

63

64

65 int stanc\_helper(int argc, const char\* argv[],

66  std::ostream\* out\_stream, std::ostream\* err\_stream) {

67  static const int SUCCESS\_RC = 0;

68  static const int EXCEPTION\_RC = -1;

69  static const int PARSE\_FAIL\_RC = -2;

70  static const int INVALID\_ARGUMENT\_RC = -3;

71

72  std::string out\_file\_name; // declare outside of try to delete in catch

73

74  try {

75

76  stan::io::cmd\_line cmd(argc,argv);

77

78  if (cmd.has\_flag("help")) {

79  print\_stanc\_help(out\_stream);

80  return SUCCESS\_RC;

81  }

82

83  if (cmd.has\_flag("version")) {

84  print\_version(out\_stream);

85  return SUCCESS\_RC;

86  }

87

88  if (cmd.bare\_size() != 1) {

89  std::string msg("Require model file as argument. ");

90  throw std::invalid\_argument(msg);

91  }

92  std::string in\_file\_name;

93  cmd.bare(0,in\_file\_name);

94  std::fstream in(in\_file\_name.c\_str());

95

96  std::string model\_name;

97  if (cmd.has\_key("name")) {

98  cmd.val("name",model\_name);

99  } else {

100  size\_t slashInd = in\_file\_name.rfind('/');

101  size\_t ptInd = in\_file\_name.rfind('.');

102  if (ptInd == std::string::npos)

103  ptInd = in\_file\_name.length();

104  if (slashInd == std::string::npos) {

105  slashInd = in\_file\_name.rfind('\\');

106  }

107  if (slashInd == std::string::npos) {

108  slashInd = 0;

109  } else {

110  slashInd++;

111  }

112  model\_name = in\_file\_name.substr(slashInd,ptInd - slashInd) + "\_model";

113  for (std::string::iterator strIt = model\_name.begin();

114  strIt != model\_name.end(); strIt++) {

115  if (!isalnum(\*strIt) && \*strIt != '\_') {

116  \*strIt = '\_';

117  }

118  }

119  }

120

121  if (cmd.has\_key("o")) {

122  cmd.val("o",out\_file\_name);

123  } else {

124  out\_file\_name = model\_name;

125  out\_file\_name += ".cpp";

126  }

127

128  if (!isalpha(model\_name[0]) && model\_name[0] != '\_') {

129  std::string msg("model\_name must not start with a number or symbol other than \_");

130  throw std::invalid\_argument(msg);

131  }

132  for (std::string::iterator strIt = model\_name.begin();

133  strIt != model\_name.end(); strIt++) {

134  if (!isalnum(\*strIt) && \*strIt != '\_') {

135  std::string msg("model\_name must contain only letters, numbers and \_");

136  throw std::invalid\_argument(msg);

137  }

138  }

139

140  stan::gm::program prog;

141  std::fstream out(out\_file\_name.c\_str(),

142  std::fstream::out);

143  if (out\_stream) {

144  \*out\_stream << "Model name=" << model\_name << std::endl;

145  \*out\_stream << "Input file=" << in\_file\_name << std::endl;

146  \*out\_stream << "Output file=" << out\_file\_name << std::endl;

147  }

148  bool valid\_model

149  = stan::gm::compile(err\_stream,in,out,model\_name,in\_file\_name);

150  out.close();

151  if (!valid\_model) {

152  if (err\_stream)

153  \*err\_stream << "PARSING FAILED." << std::endl;

154  delete\_file(out\_stream,out\_file\_name); // FIXME: how to remove triple cut-and-paste?

155  return PARSE\_FAIL\_RC;

156  }

157  } catch (const std::invalid\_argument& e) {

158  if (err\_stream) {

159  \*err\_stream << std::endl

160  << e.what()

161  << std::endl;

162  \*err\_stream << "Execute \"stanc --help\" for more information"

163  << std::endl;

164  delete\_file(out\_stream,out\_file\_name);

165  }

166  return INVALID\_ARGUMENT\_RC;

167  } catch (const std::exception& e) {

168  if (err\_stream) {

169  \*err\_stream << std::endl

170  << e.what()

171  << std::endl;

172  }

173  delete\_file(out\_stream,out\_file\_name);

174  return EXCEPTION\_RC;

175  }

176

177  return SUCCESS\_RC;

178 }

179

stan::io::cmd\_line::val

bool val(const std::string &key, T &x) const

Returns the value for the key provided.

**Definition:** cmd\_line.hpp:189

delete\_file

void delete\_file(std::ostream \*err\_stream, const std::string &file\_name)

**Definition:** stanc\_helper.hpp:55

stan::io::cmd\_line::has\_key

bool has\_key(const std::string &key) const

Return true if the specified key is defined.

**Definition:** cmd\_line.hpp:165

stan::gm::program

**Definition:** ast.hpp:795

stan::io::cmd\_line

Parses and stores command-line arguments.

**Definition:** cmd\_line.hpp:113

print\_version

void print\_version(std::ostream \*out\_stream)

**Definition:** stanc\_helper.hpp:12

stanc\_helper

int stanc\_helper(int argc, const char \*argv[], std::ostream \*out\_stream, std::ostream \*err\_stream)

**Definition:** stanc\_helper.hpp:65

compiler.hpp

stan::io::cmd\_line::has\_flag

bool has\_flag(const std::string &flag) const

Return true if the specified flag is defined.

**Definition:** cmd\_line.hpp:203

cmd\_line.hpp

stan::MINOR\_VERSION

const std::string MINOR\_VERSION

Minor version number for Stan package.

**Definition:** version.hpp:12

stan::io::cmd\_line::bare\_size

size\_t bare\_size() const

Return the number of bare arguments.

**Definition:** cmd\_line.hpp:212

stan::PATCH\_VERSION

const std::string PATCH\_VERSION

Patch version for Stan package.

**Definition:** version.hpp:15

stan::math::e

double e()

Return the base of the natural logarithm.

**Definition:** constants.hpp:86

print\_stanc\_help

void print\_stanc\_help(std::ostream \*out\_stream)

Prints the Stan compiler (stanc) help.

**Definition:** stanc\_helper.hpp:26

stan::io::cmd\_line::bare

bool bare(size\_t n, T &x) const

Returns the bare argument.

**Definition:** cmd\_line.hpp:230

stan::io::print\_help\_option

void print\_help\_option(std::ostream \*o, const std::string &key, const std::string &value\_type, const std::string &msg, const std::string &note="")

Prints single print option to output ptr if non-null.

**Definition:** cmd\_line.hpp:74

stan::MAJOR\_VERSION

const std::string MAJOR\_VERSION

Major version number for Stan package.

**Definition:** version.hpp:9

version.hpp

stan::gm::compile

bool compile(std::ostream \*msgs, std::istream &stan\_gm\_in, std::ostream &cpp\_out, const std::string &model\_name, const std::string &in\_file\_name="input")

Read a Stan model specification from the specified input, parse it, and write the C++ code for it to ...

**Definition:** compiler.hpp:37


---

[ Stan Home Page ]

*© 2011–2014,
Stan Development Team.*
